# Invasive carcinoma derived from branch duct-type IPMN may be a more aggressive neoplasm than that derived from main duct-type IPMN

**DOI:** 10.3892/ol.2013.1268

**Published:** 2013-03-21

**Authors:** TAKEHIRO OKABAYASHI, YASUO SHIMA, TAKUHIRO KOSAKI, TATSUAKI SUMIYOSHI, AKIHITO KOZUKI, TASTUO IIYAMA, YUKA TAKEZAKI, MICHIYA KOBAYASHI, ISAO NISHIMORI, YASUHIRO OGAWA, KAZUHIRO HANAZAKI

**Affiliations:** 1 Department of Surgery, Kochi Health Sciences Center, Kochi 781-8555;; 2 Departments of Gastroenterology and Hepatology, Kochi Medical School, Kochi University, Nankoku, Kochi 7838505, Japan; 3 Biostatistics, Kochi Medical School, Kochi University, Nankoku, Kochi 7838505, Japan; 4 Surgery, Kochi Medical School, Kochi University, Nankoku, Kochi 7838505, Japan; 5 Radiology, Kochi Medical School, Kochi University, Nankoku, Kochi 7838505, Japan

**Keywords:** intraductal papillary mucinous neoplasm, prognosis, SMAD4, TGF-β, surgery, management

## Abstract

The present study aimed to evaluate the long-term follow-up results of patients with intraductal papillary mucinous neoplasm (IPMN) and to estimate the degree of IPMN malignancy based on pathological and molecular features of resected specimens. The detection rate of IPMN has increased over the last decade; however, the management of this neoplasm remains controversial. This is particularly so for branch duct-type IPMN, which carries a high potential for malignancy and risk of recurrence. We retrospectively reviewed a single institution’s prospective pancreatic resection database to identify IPMN patients who underwent pancreatectomy with curative intent. The clinicopathological variables of 100 patients resected for IPMN were analyzed with a detailed review of histopathological results (borderline lesions, non-invasive carcinoma and invasive carcinoma) to determine the grade of IPMN malignancy based on transforming growth factor (TGF)-β/SMAD4 signaling. The incidence of malignant change was significantly higher in patients with main duct-type IPMN (69.7%) compared with branch duct-type IPMN cases (17.9%). However, patients with an invasive carcinoma had a significantly worse outcome if it was derived from branch duct-type IPMN compared with those derived from main duct-type IPMN, and TGF-β mRNA expression was significantly increased in the former patient group. Immunohistochemistry also showed higher numbers of SMAD4-positive cells in patients with carcinoma derived from branch duct-type IPMN. Our results demonstrated that invasive carcinoma derived from branch duct-type IPMN is more aggressive than that derived from main duct-type IPMN, once invasive morphological change takes place. Determining TGF-β and/or SMAD4 status at initial diagnosis may be useful for stratifying IPMN patients into treatment regimens.

## Introduction

Intraductal papillary mucinous neoplasm (IPMN) of the pancreas is a relatively new entity that is being diagnosed with increasing frequency (1). IPMN is characterized by intraductal proliferation of neoplastic mucinous cells, which show varying degrees of atypia and usually form papilla that lead to cystic dilatation of pancreatic ducts and subsequently to clinically detectable masses (2). IPMN has been established as a precursor of pancreatic adenocarcinoma via the hyperplasia, dysplasia and invasive carcinoma sequence. However, the incidence of this full progression varies greatly with the site of origin (main duct or branch duct), and IPMN grade may be difficult to distinguish clinically, particularly in the absence of surgery (3). Therefore, all IPMN cases without exception should be considered potentially malignant (4,5).

In 2004, an international conference defined the International Consensus Guidelines (ICG) for selecting patients for immediate surgery or a surveillance strategy (6). Main pancreatic duct-type IPMN cases are frequently malignant and the optimal management of such tumors is now widely acknowledged to be resection of the lesion, provided that the patient is fit enough for this intervention (7–10). However, it is has been suggested that branch duct-type IPMN lesions >3 cm in size and with suspicious radiological changes, including the presence of mural nodules inside the cystic lesion, a dilated main pancreatic duct or positive cytological findings, should be recommended for surgical resection due to their higher risk of malignant potential. However, this is a rapidly evolving field and there are a significant number of areas where there remains no consensus (11).

Early studies of IPMN suggested that patients resected for branch duct-type IPMN had a more benign neoplasm compared with those with main duct-type IPMN (12-14). Short- and mid-term follow-up (median, <5 years) studies led to the conclusion that morphological changes are rare events with branch duct-type IPMN; however, the long-term evolution and/or biological behavior of this tumor subgroup remain unknown (15,16). Establishing such data on branch duct-type IPMN is thus of great importance for the patients and clinical management teams. SMAD4 is a tumor suppressor gene on chromosome 18q21.1 that is inactivated in >50% of pancreatic malignancies, and SMAD4 protein overexpression suppresses cell proliferation in malignant pancreatic neoplasms (17,18). However, it remains unclear how SMAD4 is retained in intraductal lesions, while its loss is frequently observed in invasive IPMNs.

The purpose of this study was to characterize the clinicopathological features of patients with branch duct-type IPMN resected at our institution with detailed examination of histopathological and molecular investigations, and to compare the outcomes of these patients with those with main duct-type IPMN. Furthermore, we discuss the malignant potential of invasive intraductal papillary mucinous carcinoma (IPMC) derived from branch duct-type IPMN based on our analysis of the TGF-β/SMAD4 pathway.

## Patients and methods

### Patients, surgery and pathological classification

This is a study of prospectively collected, retrospectively analyzed data. The diagnosis of IPMN was suspected following imaging and endoscopic analysis and was confirmed by pathological analysis. We retrospectively reviewed the surgical pathology database of Kochi Medical School to identify patients who underwent resection for IPMN. Case selection was restricted to patients who underwent resection in or after 2000, as since then all clinical diagnoses of IPMN of the pancreas were evaluated using standardized diagnostic modalities, including computed tomography (CT), magnetic resonance imaging (MRI), endoscopic retrograde cholangiopancreatography (ERCP) and in particular endoscopic ultrasonography (EUS). All IPMN patients who had undergone a pancreatic resection in the Department of Surgery at Kochi Medical School between January 2000 and December 2011 were included in this study.

The indication for resection or surveillance was verified *a posteriori* for all patients in accordance with the ICG (6). All patients with main duct-type IPMN, symptomatic branch duct-type IPMN or asymptomatic branch duct-type IPMN >30 mm in size and/or with mural nodules and/or a dilated main pancreatic duct were referred for immediate surgical resection. Patients with asymptomatic branch duct-type IPMN <30 mm in size without mural nodules or dilated main pancreatic duct were placed under careful monitoring and surveillance. Patients placed under surveillance underwent clinical examination, laboratory tests, including for carcinoembryonic antigen (CEA) expression and carbohydrate antigen (CA) 19-9 serum levels, as well as CT, MRI and EUS every 6 months for 2 years, and yearly thereafter. Surgery was also performed when cysts showed significant growth or when suspicion of malignancy was increased, even if the original size of the cystic pancreatic lesion was <30 mm (19).

The diagnosis was validated on the basis of the histological findings in a surgical specimen, or the outcome of surveillance. The lesions were classified into three categories according to the World Health Organization classification: slight dysplasia and intraductal papillary mucinous adenoma, moderate dysplasia or borderline malignancy (borderline IPMN), severe dysplasia or IMPC *in situ* (non-invasive IPMC) and invasive carcinoma (invasive IPMC). When more than one pathological type was present, the tumor was classified according to the worst lesion present.

The study was approved by the ethics committee of Kochi Medical School. Written informed consent was obtained from the patients.

### Clinical pre- and post-operative evaluation in patients with IPMN of the pancreas

Medical records were reviewed retrospectively for the following information: patient characteristics, clinical history, physical examination, laboratory investigations, surgical management, pathology examinations and post-operative course. Any history of a previous extra-pancreatic neoplasm or ordinary pancreatic carcinoma was investigated thoroughly. Body mass index (BMI) was calculated as weight (kg) divided by height squared (m^2^). A self-administered questionnaire was used to determine the smoking and drinking habits of all IPMN patients. Data of patient outcomes were obtained through retrospective review of a prospectively maintained pancreatic resection database, electronic hospital charts and medical records.

### RNA isolation and real-time reverse transcription polymerase chain reaction (RT-PCR) for TGF-β

Representative formalin-fixed and paraffin-embedded (FFPE) sections of all IPMN tumors were collected from the surgical pathology archives. In the present study, we extracted RNA from the samples of both main and branch duct-type IPMN of the pancreas using the RNeasy FFPE Kit (Qiagen, Hilden, Germany) (20). Total RNA yield and purity was estimated by UV spectroscopy (Nanodrop ND-1000 Spectrophotometer; Nanodrop Technologies, Wilmington, DE, USA) and RNA quality was assessed on an Agilent 2100 Bioanalyzer (Agilent Technologies, Santa Clara, CA, USA). First-strand cDNA synthesis was then performed with 2.5 *μ*g total RNA using the superscript first-strand synthesis system for RT-PCR (Invitrogen, Carlsbad, CA, USA) according to the manufacturer’s instructions. We measured the expression of TGF-β with normalization as previously described (21). Real-time RT-PCR was carried out using the Power SYBR-Green PCR Master mix (Applied Biosystems, Warrington, UK) as described previously (21). Primers used for PCR were as follows: TGF-β-forward: 5′-gcagcacgtggagctgta-3′; TGF-β-reverse: 5′-cagccggttgctgaggta-3′. PCR conditions for all genes were as follows: 95°C initial activation for 10 min followed by 40 cycles of 95°C for 15 sec and 60°C for 60 sec, and fluorescence determination at the melting temperature of the product for 20 sec on an ABI PRISM 7000 (Applied Biosystems).

### Immunohistochemistry for SMAD4 protein

The streptavidin-biotin-peroxidase method with the Dako kit (Carpinteria, CA, USA) was used to detect SMAD4 protein expression on three serially cut representative sections. Following inactivation of endogenous peroxidase and blocking of nonspecific antibody binding, the specimens were treated with biotinylated antibodies specific for SMAD4 (1:100, Q13485, Epitomics, Abcam, Cambridge, MA, USA) at 4°C overnight. Subsequently, sections were incubated with the streptavidin-biotin-peroxidase complex reagent for 30 min at room temperature. Diaminobenzidine tetrahydrochloride was used as the chromogen and hematoxylin was used for counterstaining.

### Statistical analysis

Continuous variables are presented as mean ± SD. Dichotomous variables are presented as both number and percentage values. Data were analyzed using Student’s t-test (two-tailed), with dichotomous variables analyzed by the χ^2^ test (two-tailed) or Fisher’s exact test (two-tailed) by a biostatistics specialist, as appropriate. Survival probabilities were determined using the Kaplan-Meier method and compared using the log-rank test. Survival analysis excluded patients who died in the 30-day postoperative period. Cause of mortality was not available for all patients, so only overall survival was calculated. P<0.05 was considered to indicate a statistically significant result. All analyses were performed using SPSS^®^ (SPSS, Inc., Chicago, IL, USA).

## Results

### Patient characteristics

Of the 100 patients enrolled in the present study, 33 (33.0%) were found to have main duct-type IPMN (69.7% male) and 67 (67.0%) had branch duct-type IPMN (68.7% male; Table I). Mortality 30 days after pancreatic resection was 1.0%; the one patient who died had borderline IPMN of the head of the pancreas and underwent pancreaticoduodenectomy. However, septic shock developed as a consequence of bacterial endocarditis and the patient died on the 14th post-operative day. There were no significant differences in age, gender or BMI between patients with main duct-type IPMN or with branch duct-type IPMN. As shown in Table I, patients with main duct-type IPMN had a significantly higher incidence of abdominal pain (51.5 vs. 20.9%; P=0.002), while those with branch duct-type IPMN had a significantly higher incidence of enlarged tumor growth (37.3 vs. 12.1%; P=0.017). There were no significant differences in past medical history, including diabetes mellitus and hypertension incidence, alcohol consumption and cigarette smoking, between the groups. Among the 100 patients with IPMN, 12 patients (12.0%) had an ordinary pancreatic carcinoma; 2 cases (6.1%) in patients with main duct-type IPMN and 10 cases (14.9%) in patients with branch duct-type IPMN. Notably, patients suffering from IPMN had the highest incidence of malignancy compared with their family history in the two groups; 57.6% in patients with main duct-type IPMN and 52.2% in patients with branch duct-type IPMN (Table I). Indeed, 29 patients with IPMN (29.0%) had a past medical history of other neoplasms (Table I).

### Comparison of clinicopathological findings

Table II demonstrates the clinicopathological variables and tumor and treatment characteristics of the 100 patients who underwent surgical resection of IPMN. There were no significant differences in expression of the pre-operative tumor markers CEA and CA 19-9 between main and branch duct-type IPMN cases. Immediate surgery was performed in 81.8% of patients with main duct-type IPMN and in 53.7% of patients with branch duct-type IPMN (P=0.027), although postoperative follow-up periods were significantly longer in the latter group of patients (median, 1.6 years; range, 1–2 years) than in the former (median, 3.6 years; range, 1–10 years; P=0.021). There was no significant difference in the surgical procedures between the groups. In total, pancreaticoduodenectomy was performed in 48 patients, distal pancreatectomy with splenectomy in 34 patients and 6 patients underwent a total pancreatectomy. Notably, minimally invasive pancreatic surgery was performed in only 10 patients with branch duct-type IPMN, comprising duodenum-preserving pancreatic head resection in 5 patients, central pancreatectomy in 3 patients, inferior head resection in 1 patient and spleen-preserving distal pancreatectomy in 1 patient. The incidence of malignant change in patients with main duct-type IPMN (69.7%) was significantly higher than that in patients with branch duct-type IPMN (17.9%), as expected. Of the 33 main duct-type IPMN cases, 10 (30.3%) were diagnosed as borderline IPMN, 14 (42.4%) were non-invasive IPMC and 9 (27.3%) were invasive IPMC. Of the 67 branch duct-type IPMN cases, 55 (82.1%) were borderline IPMN, 3 (4.5%) were non-invasive IPMC and 9 (13.4%) were invasive IPMC, with 10 of these 12 malignant IPMC patients exhibiting mural nodules in cystic lesions of the pancreas that were found to be malignant on pathological findings obtained from surgical resection. However, the remaining 2 patients also had a malignant neoplasm derived from branch duct-type IPMN, although the cystic lesions of the pancreas had no mural nodules.

### Survival

**Figure 1:**
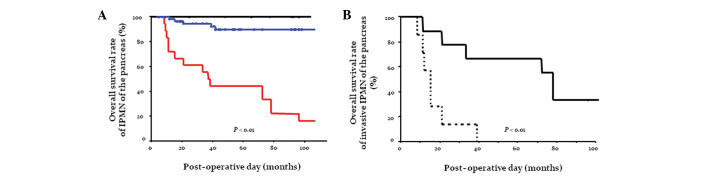
(A) Actuarial survival curves following resection of IPMN based on pathological type. Patients with an invasive IPMC had a significantly worse outcome compared with those with borderline or non-invasive IPMC. The rate of cumulative 5-year survival following curative resection of invasive adenocarcinoma derived from IPMN was 44.4% (median survival, 37.0 months). Overall survival rate of borderline IPMN of the pancreas (%) (blue line), non-invasive IPMC (black line) and invasive IPMC (red line). (B) Cumulative survival curves following resection of invasive adenocarcinoma derived from IPMN. Patients with an invasive adenocarcinoma derived from branch duct-type IPMN had a significantly worse outcome compared with those with invasive adenocarcinoma derived from main duct-type IPMN. The 5-year survival rates following curative resection of invasive adenocarcinoma were 0.0% (median survival, 15.0 months) for those tumors derived from branch duct-type IPMN and 66.7% (median survival, 78.0 months) for tumors derived from main duct-type IPMN. Overall survival rate of invasive IPMC derived from main duct-type IPMN of the pancreas (%) (solid line) and those derived from branch duct-type IPMN of the pancreas (broken line). IPMN, intraductal papillary mucinous neoplasm; IPMC, intraductal papillary mucinous carcinoma.

Overall survival following resection was analyzed in patients with IPMN (n=87) after excluding patients who died in the 30-day post-operative period (1/100, 1.0%) and those suffering from ordinary pancreatic carcinoma (12/100, 12.0%). Patient follow-up as of December 2011 ranged from 4 to 196 months, with a median of 54 months (mean, 63.1 months). In general, patients with an invasive IPMC had a significantly worse outcome compared with those with borderline or non-invasive IPMC (Fig. 1A). The cumulative 5-year survival rate following curative resection of invasive adenocarcinoma derived from IPMN was 44.4% (median survival, 37.0 months), whereas borderline IPMN and non-invasive IPMC behaved more favorably. The cumulative survival following curative resection in patients with adenocarcinoma derived from IPMN was then sub-analyzed. Notably, patients with an invasive adenocarcinoma derived from main duct-type IPMN had a significantly better outcome (66.7% surviving at 5 years; median survival, 78.0 months) than those with invasive adenocarcinoma derived from branch duct-type IPMN (0.0% surviving at 5 years; median survival, 15.0 months; Fig. 1B).

### Evaluation of TGF-β/SMAD4 signaling in patients with IPMN

**Figure 2:**
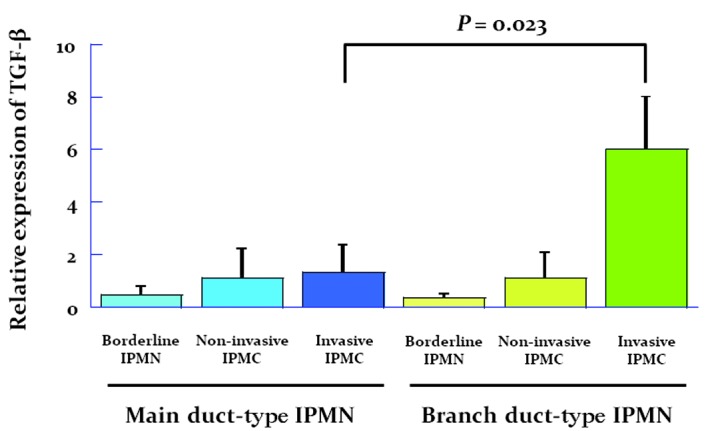
Relative expression of TGF-β in patients with IPMN. Real-time RT-PCR was employed to measure the expression levels of TGF-β. The results are expressed in arbitrary units as a ratio of the target gene transcripts to TGF-β transcripts. Results are the mean ± SD, unpaired t-test. The TGF-β mRNA expression was significantly increased in patients with adenocarcinoma derived from branch duct-type IPMN compared with patients with borderline IPMN, and especially with adenocarcinoma derived from main duct-type IPMN (P=0.023). TGF-β, transforming growth factor-β; IPMN, intraductal papillary mucinous neoplasm; RT-PCR, reverse-transcription polymerase chain reaction.

**Figure 3:**
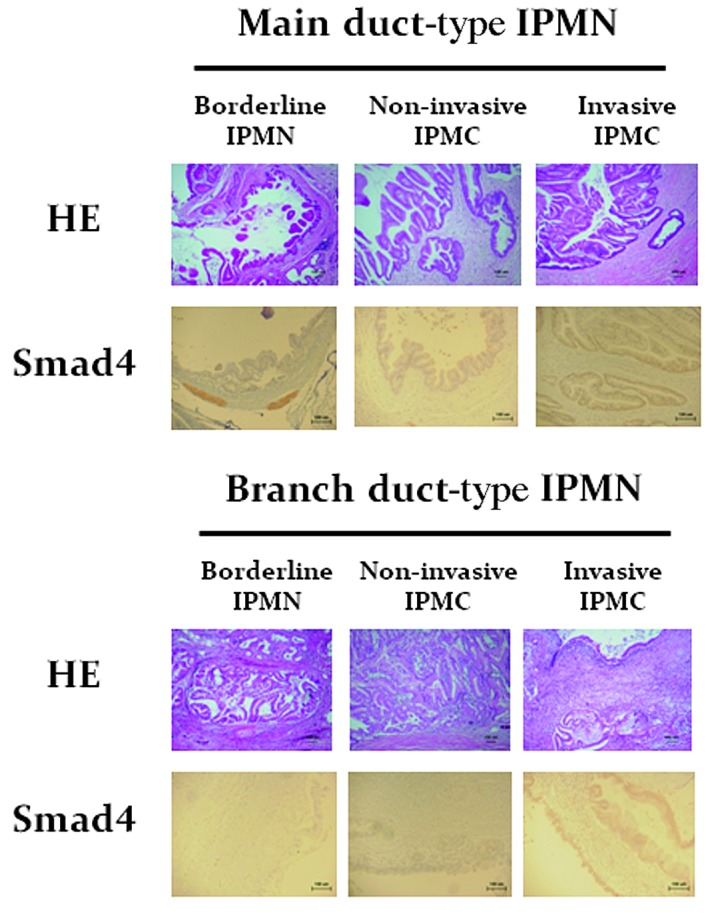
Pathological findings and immunochemistry for SMAD4 staining of IPMN. Histological sections from patients with IPMN were stained by HE and with an antibody against SMAD4. Magnification, ×100. Immunohistochemistry demonstrated that the number of SMAD4-positive cells was increased in patients with adenocarcinoma derived from branch duct-type IPMN. IPMN, intraductal papillary mucinous neoplasm; HE, hematoxylin-eosin; IPMC, intraductal papillary mucinous carcinoma.

The overall survival in patients with an invasive adenocarcinoma derived from branch duct-type IPMN was significantly worse than in those patients with invasive adenocarcinoma derived from main duct-type IPMN. We therefore examined TGF-β/SMAD4 signaling in all patients. Fig. 2 shows the expression in arbitrary units as a ratio of the target gene transcripts to TGF-β transcripts by real time RT-PCR. Notably, the mRNA expression of TGF-β was significantly increased in patients with adenocarcinoma derived from branch duct-type IPMN compared with patients with borderline IPMN and especially with those with adenocarcinoma derived from main duct-type IPMN. Immunohistochemical staining for SMAD4 protein in tissue sections of the pancreas obtained from patients with IPMN showed that the number of SMAD4-positive cells was increased in patients with adenocarcinoma derived from branch duct-type IPMN (Fig. 3).

## Discussion

In this retrospective study, we found that invasive carcinoma derived from branch duct-type IPMN was more aggressive than that derived from main duct-type IPMN, once invasive morphological change was apparent. This study also clarified the progression pattern of TGF-β/SMAD4 signaling in IPMNs.

As observed in previous studies, 55 (82.1%) of the 67 patients in this study with branch duct-type IPMN had a benign neoplasm at the time of initial pre-operative surgical indication (12–14). The most noteworthy finding in the present study is, therefore, that patients with invasive carcinoma derived from branch duct-type IPMNs, excluding patients with ordinary pancreatic adenocarcinoma, had an extremely poor prognosis, whereas patients with malignant IPMNs derived from main duct-type IPMN had a relatively better prognosis following surgical treatment. In recent years, an increasing number of studies concerning follow-up clinical and imaging data for branch duct-type IPMN have indicated that few such patients develop malignancy (16,22,23). A previous study reported that deletion of DPC4 (a tumor-suppressor gene) increased aggressive cancer and decreased survivability (24). However, our results with regard to the increased invasive nature of branch duct-type IPMN are in apparent contradiction with those of the previous study. Furthermore, our results also indicate that ICG was an unsatisfactory method to select patients with malignant IPMN, prompting us to challenge the molecular analysis of TGF-β/SMAD4 signaling in IPMN (25).

TGF-β is a potent inhibitor of epithelial cell growth and survival through modulating the expression of cell cycle regulators and activating apoptosis, although these effects are highly dependent on cellular context (26). However, TGF-β enhances the malignant growth of certain established epithelial tumors, promoting tumor cell proliferation, migration and the epithelial-to-mesenchymal transition, which is a process by which advanced carcinomas acquire a highly invasive, undifferentiated and metastatic phenotype (27). Therefore, TGF-β signaling may have biphasic stage-specific effects: inhibiting carcinoma initiation while promoting the high-grade advancement and dissemination of established tumors (28). In the present study, real-time RT-PCR revealed significantly increased mRNA expression of TGF-β in patients with carcinoma derived from branch duct-type IPMN, and patients expressing SMAD4 had significantly worse outcomes. TGF-β/SMAD4 signaling may therefore have pleiotropic and context-dependent roles in IPMN and the present study suggested that determining the TGF-β and/or SMAD4 status of a tumor at initial diagnosis may be of value for stratifying patients into treatment regimens (surgical management vs. conservative follow-up).

Pancreatic surgery is burdened by significant morbidity and mortality, even at specialized centers (29). Resecting premalignant or potentially premalignant lesions affords an unprecedented opportunity to perform a greater number of minimally invasive pancreatic surgeries. However, the indication of such surgery for pancreatic neoplasms remains controversial and is not described in the ICG. In the present study, minimally invasive pancreatic surgery was performed only in those patients with borderline IPMN, and these patients had no recurrence. The most important consideration is not allowing patients with borderline or non-invasive IPMN to succumb to recurrent disease following curative surgery, even if patients with invasive carcinoma arising in the setting of an IPMN appear to have a more favorable outcome than patients with resectable ordinary pancreatic carcinoma (8,30). Therefore, surgeons should select IPMN patients for minimally invasive pancreatic surgery based on an array of histological features and a spectrum of biological behaviors, as optimal diagnosis and risk stratification are often challenging.

The prognosis and recurrence rate of IPMN depend mainly on tumor invasiveness and the type of duct involved. The recurrence rate is higher for invasive lesions of the branch duct, and such lesions must therefore be treated surgically as soon as feasibly possible, similar to classic pancreatic adenocarcinoma. Advances in imaging technologies have increased the number of diagnoses of asymptomatic lesions, thus more stringent and careful criteria should be included in the ICG to increase their specificity and define malignancy risk pre-operatively. As it stands, the ICG definition of differential clinical diagnosis for IPMN of the pancreas is unsatisfactory with regard to malignant status. Indeed, patients with IPMN, invasive or not, should be submitted for lifetime follow-up checking for recurrence in the remnant pancreas and for associated cancers.

## Figures and Tables

**Table I:** Patient characteristics.

Characteristics	Main duct-type IPMN (n=33)	Branch duct-type IPMN (n=67)	P-value
Patient details			
Age (years), mean ± SD	68.7±6.8	66.9±10.8	0.381
Male (%)	69.7	68.7	0.916
Body mass index, mean ± SD	22.8±3.8	21.9±3.0	0.187
Presenting sign/symptoms (%)			
Abdominal pain	51.5	20.9	0.002
Tumor enlarged	12.1	37.3	0.017
Group examination	24.2	22.4	0.964
Past medical history (%)			
Diabetes mellitus	48.5	40.3	0.437
Hypertension	50.0	38.8	0.594
Cigarette smoking	63.6	67.2	0.726
Alcohol consumption	66.7	56.7	0.340
Other neoplasms	36.4	25.4	0.255
Suffering from pancreatic cancer (%)	6.1	14.9	0.157
Family history of malignancy (%)	57.6	52.2	0.615

IPMN, intraductal papillary mucinous neoplasm.

**Table II:** Comparison of clinicopathological findings between patients with main duct-type IPMN and branch duct-type IPMN.

Characteristic	Main duct-type IPMN (n=33)	Branch duct-type IPMN (n=67)	P-value
Tumor marker, blood chemistry			
Carcinoembryonic antigen (ng/ml)	3.0±3.0	2.7±2.1	0.566
Carbohydrate antigen 19–9 (U/ml)	55.5±88.0	48.1±182.7	0.850
Surgical period			
Immediately (%)	81.8	53.7	0.027
Follow-up (%)	18.2	46.3	
Follow-up, median years (range)	1.6 (1–2)	3.6 (1–10)	0.021
Surgical procedure (n)			
Total pancreatectomy	4	2	0.143
Pancreaticoduodenectomy	17	31	
Distal pancreatectomy	10	24	
Minimal invasive surgery	0	10	
Pathology			
Adenoma	10	55	
Non-invasive carcinoma	14	3	0.001
Invasive carcinoma	9	9	

IPMN, intraductal papillary mucinous neoplasm.
